# A Longitudinal Study on Attention Development in Primary School Children with and without Teacher-Reported Symptoms of ADHD

**DOI:** 10.3389/fpsyg.2017.00655

**Published:** 2017-05-16

**Authors:** Elisabet Suades-González, Joan Forns, Raquel García-Esteban, Mónica López-Vicente, Mikel Esnaola, Mar Álvarez-Pedrerol, Jordi Julvez, Alejandro Cáceres, Xavier Basagaña, Anna López-Sala, Jordi Sunyer

**Affiliations:** ^1^Center for Research in Environmental Epidemiology, Barcelona Institute for Global HealthBarcelona, Spain; ^2^Department of Experimental and Health Sciences, Pompeu Fabra UniversityBarcelona, Spain; ^3^Learning Disabilities Unit (UTAE), Neuropediatrics Department, Hospital Sant Joan de Déu, University of BarcelonaBarcelona, Spain; ^4^CIBER Epidemiología y Salud PúblicaBarcelona, Spain; ^5^Hospital del Mar Medical Research InstituteBarcelona, Spain; ^6^Environmental Health Department, Harvard T.H. Chan School of Public HealthBoston, MA, USA

**Keywords:** attention network test, attention, children, neurodevelopment, multilevel analysis, population study, longitudinal study

## Abstract

**Background:** Prospective longitudinal studies are essential in characterizing cognitive trajectories, yet few of them have been reported on the development of attention processes in children. We aimed to explore attention development in normal children and children with attention deficit and hyperactivity disorder (ADHD) symptoms in a repeated measures design using the attention network test (ANT).

**Methods:** The population sample included 2,835 children (49.6% girls) aged 7–11 years from 39 schools in Barcelona (Catalonia, Spain) who performed the ANT four times from January 2012 to March 2013. According to teacher ratings, 10.5% of the children presented ADHD symptoms. We performed multilevel mixed-effects linear regression models, adjusting for school and individual, to test the effects of age-related growth on the ANT networks: alerting, orienting and executive attention, and three measurements related to attentiveness: median of hit reaction time (HRT), hit reaction time standard error (HRT-SE) and variability.

**Results:** We observed age-related growth in all the outcomes, except orienting. The curves were steeper at the younger groups, although for alertness the improvement was further at the oldest ages. Gender and ADHD symptoms interacted with age in executive attention, HRT and variability. Girls performed better in executive attention at young ages although boys reached females at around 10 years of age. For HRT, males showed faster HRT. However, girls had a more pronounced improvement and reached the levels of boys at age 11. Children with ADHD symptoms had significant differences in executive attention, HRT and variability compared to children without ADHD symptoms.

**Conclusions:** We detected an ongoing development of some aspects of attention in primary school children, differentiating patterns by gender and ADHD symptoms. Our findings support the ANT for assessing attention processes in children in large epidemiological studies.

## Introduction

Attention is a complex cognitive function involving different processes like selectively attending to specific stimuli, focusing for prolonged periods, or regulating and monitoring of actions (Anderson, 2002). It is a basic function that precedes higher-level cognitive abilities (e.g., executive functions or memory). Furthermore, attention underlies our awareness of the world and the voluntary regulation of our thoughts and feelings (Posner and Rothbart, 2007). The evaluation of attention in children is important because of its implications on learning, academic achievement and social functioning (Spira and Fischel, 2005).

Posner and Petersen were the first to propose a framework that divides attention into three functionally and anatomically separate networks: alerting, orienting and executive attention (Posner and Petersen, 1990; Fan et al., 2005). Alerting is the ability to produce and maintain optimal vigilance and performance during tasks (Petersen and Posner, 2012); the brain areas implicated are locus coeruleus, right frontal and parietal cortex. Orienting involves shifting attention to endogenous or exogenous cues (Corbetta et al., 1998; Posner et al., 2006); it involves parietal sites and frontal eye fields. Executive attention involves detecting and resolving conflict among responses, error detection and response inhibition (Bush et al., 2000); anterior cingulate cortex and prefrontal areas are involved in this network.

Following Posner and Petersen's model, Fan et al. developed the attention network test (ANT) to assess the three attentional networks (Fan et al., 2002). The ANT combines the flanker task (Eriksen and Eriksen, 1974), a widely used measure of executive control processes, with the use of cues entailing just alertness or alertness plus orienting information.

Rueda et al. adapted the adult ANT with a format and design more attractive for use with children from 4 years of age (Rueda et al., 2004). It is a rapid and easy-to-apply computerized test that has been validated as a tool for measuring attention processes in large-scale epidemiological studies (Forns et al., 2014) in which efficiency, precision and objectivity of data collection from using a computerized format is a major benefit.

Differences between age-group means in children, adolescents and adults have been encountered in attention processes using the ANT according to previous cross-sectional studies (Mezzacappa, 2004; Rueda et al., 2004; Gupta and Kar, 2009; Federico et al., 2017). Attention starts to develop early in infancy (e.g., exogenous alertness, orienting to external cues). Then, by the end of the first year of life, a more voluntarily controlled attention emerges. During childhood, the three networks continue developing and showing improvement in the endogenous sustained attention, the reorienting of attention, and the inhibitory control. Furthermore, the executive attention network, which depends on the maturation of the prefrontal cortex, shows a longer development into adolescence (Welsh and Pennington, 1988; Rueda et al., 2004, 2015; Konrad et al., 2005; Romine and Reynolds, 2005; Amso and Johnson, 2006). This is in relation to the many neurophysiological changes that occur in the brain (proliferation, migration, differentiation, synaptogenesis, myelination, and apoptosis) from the embryonic period through adolescence (Rice and Barone, 2000). In addition, there are genetic and environmental contributions to these changes on the developing brain, and therefore the attention circuits, during this period (Amso and Scerif, 2015). Gender can also impact attentional performance and although very few studies have focused on gender differences, girls seem to be more-advantaged (Pascualvaca et al., 1997; Klenberg et al., 2001). Furthermore, abnormal attention development is a symptom of attention deficit hyperactivity disorder (ADHD) in relation to structural and functional brain pathological changes (Biederman and Faraone, 2005). Cross-sectional studies that evaluated the three attentional systems in children with ADHD using the ANT, encountered deficits in the alerting and executive attention networks (Johnson et al., 2008; Gupta and Kar, 2009; Mullane et al., 2011; Casagrande et al., 2012). Specifically, children with ADHD showed lower levels of alertness, consistent with previous theories emphasizing difficulties in arousal regulation in relation to deficits in sustained attention (Russell et al., 2006; Johnson et al., 2007). Furthermore, children with ADHD were more vulnerable to the interference demands, and indeed, response inhibition has been described as the primary deficit in ADHD (Barkley, 1997). To our knowledge, there are no previous longitudinal studies quantifying intra-individual changes over time using the ANT. Prospective longitudinal studies on cognitive function are crucial for understanding typical developmental trajectories, confirming theories of neurodevelopment, and detecting the impact of environmental and social risk factors on cognition in epidemiological studies (Amso and Scerif, 2015; Sunyer et al., 2015; López-Vicente et al., 2016). In contrast to cross-sectional designs, longitudinal studies control for intra-individual differences across time as well as cohort effects and age-related differences in rate of change (Sliwinski and Buschke, 1999; Kraemer et al., 2000). In counterpoint, the magnitude of the practice effects due to repeated testing is a main concern (Dikmen et al., 1999). Ishigami et al. explored the stability, isolability, robustness and reliability of the ANT when administered repeatedly to older adults and patients with multiple sclerosis. Despite some practice effects in alerting and executive control, the network scores were robust against practice (Ishigami and Klein, 2011; Ishigami et al., 2013).

We therefore studied the performance of the ANT in a large longitudinal cohort of 7–11 year old children to detect patterns in attention growth. We focused on this age range for several reasons. First, and as mentioned above, some aspects of attention continue to improve throughout middle and late childhood. Second, the feasibility for the administration of the test to the children in their normal class groups (e.g., understanding instructions, ability to work almost autonomously, assuring a prolonged isolation during the execution of the test). Finally, primary school children from 7 years of age were selected in order to increase accuracy of identification of ADHD symptomatology (Applegate et al., 1997). For the current study, in addition to the original three attention networks based on the Posner and Petersen (1990) model, we also analyzed three other measures based on the Mirsky model of attention (Mirsky et al., 1991) and specifically, the *Focus* component which is related to attentiveness (Egeland and Kovalik-Gran, 2010). For the calculation of these scores we followed the formulas of the Continuous Performance Test (CPT), a widely used computerized measure of different aspects of attention (Conners and Staff, 2000; Oberlin et al., 2005; Adólfsdóttir et al., 2008). We particularly aimed to study the trajectories of attention in a large longitudinal cohort of children using the ANT in a repeated measures design. Furthermore, we examined potential differences between boys and girls, and the role of ADHD symptoms in attention development. Based on the literature, we expect that: (1) the oldest children will show an improvement in alerting; (2) children will show a continued development in executive attention; (3) girls will show an advantage over boys and (4) children with ADHD symptoms will show a delayed developmental pattern in alerting, executive attention and measures related to attentiveness.

## Materials and methods

### Participants

This study is part of the BREATHE (BRain dEvelopment and Air polluTion ultrafine particles in scHool childrEn) project, which aims to assess the association between air pollution in schools and the cognition and behavior of children. The BREATHE project was conducted from January 2012 to March 2013 in 36 schools in Barcelona and 3 in Sant Cugat del Vallès, a smaller city near Barcelona (Catalonia, Spain). The students from these 39 schools in 2nd, 3rd, and 4th primary grades were invited to participate via mail and/or project presentations in the schools. The total number of schoolchildren enrolled for the study was 2,904 (59% response rate). Seven subjects were excluded from the analysis due to mental, motor or sensory impairment reported by the school. The final sample size at the beginning of the study was of 2,835 children (49.6% girls) aged from 7 to 11 years (*M* = 8.6; *SD* = 0.88). The number of participants in session 1 was 2,597, 2,705 in session 2, 2,621 in session 3, and 2,546 in session 4.

All parents or legal guardians signed the informed consent approved by the Ethical Committee of the IMIM-Parc Salut Mar.

### Instruments

#### Neuropsychological testing

The tool used to assess the attention domain was the original computerized child ANT (Rueda et al., 2004). In this version, a row of five yellow fish appearing either above or below a fixation point is presented. Children are invited to “feed” the central fish as quickly as possible by pressing either the right or the left arrow key depending on the direction in which the target fish is pointing while ignoring the flanker fish, which point in either the same (congruent) or opposite (incongruent) direction than the middle fish. The target is preceded by visual signals that inform either about the upcoming of the target only (alerting cue) or about the upcoming of the target as well as its location (orienting cue) (Rueda et al., 2012). Each correct answer is followed by a simple animation sequence (the target fish blowing bubbles) and a recorded sound (“woo hoo!”). Incorrect responses are followed by a single tone and no animation of the fish (Rueda et al., 2004).

A session of the ANT consisted of 16 practice trials and four experimental blocks of 32 trials in each (128 trials in total). Each trial represented one of 8 conditions in equal proportions: two Flanker Congruency (congruent and incongruent) × four Cue Type (no cue, central cue, double cue and spatial cue).

Reaction time (RT) measures of the three attention networks were calculated using RTs associated with a correct response. RTs shorter than 100 ms were rejected from the RT calculations because of physiological implausibility implying that such a response is perseverative or anticipatory (Conners and Staff, 2000). In addition to alerting, orienting and executive attention networks, three measures related to attentiveness (HRT, HRT-SE, and variability) were calculated following the formulas of the CPT (Conners and Staff, 2000). Definition and calculations of the measures are summarized in Table 1. Measures of performance speed were analyzed since they were reported as the most appropriate for assessing change in attentional function in children (Mollica et al., 2005). Lower scores indicate more efficiency in all the measures.

**Table 1:** **Definitions and calculations of the ANT outcomes**.

**Outcomes**	**Definitions and calculations**
**ATTENTION NETWORKS**	
Alerting	RT for No cue – RT for Double Cue trials
Orienting	RT for Central Cue – RT for Spatial Cue trials
Executive attention	RT for Incongruent – RT for Congruent trials
**MEASURES RELATED TO ATTENTIVENESS**	
HRT	Median RT for correct responses
HRT-SE	Standard error of RT for correct responses. Measure of response speed consistency
Variability	Standard deviation of the 4 standard error values calculated for each block. Measure of intra-individual variability

*RT, Reaction Time, SE, Standard Error*.

Testing lasted no more than 15 minutes. We followed a strict protocol in order to minimize measurement error: administration of the test was in a quiet and spacious room in the school; children wore headphones in order to avoid noise disturbances; groups were of 10–20 students and there was a trained examiner for every 3–4 children; sufficient distance between children minimized interaction among them; and instructions were always explained following the same structure and by the same examiner. Finally, we also analyzed the influence of environmental factors during task performance, such as day of the week, season, noise, weather, time of the day, and quality of the session (Ballard, 1996). The inclusion of environmental factors as confounders did not change the results, except for alerting (Table S1).

#### Data acquisition

As part of the BREATHE project, a 1-year follow-up with four repeated measures of the attention domain was conducted in the schools, in children in 2nd, 3rd, and 4th grades (aged 7–10 years at baseline). Most children performed the ANT four times (76%). In the cases where an assessment was missed (e.g., sickness), for the data analysis we considered the temporal order of the school sessions, not the children's “real” attendance to each session. For instance, a child who missed session 2, session 3 was still considered his session 3. The intervals between the test administrations were on average 3 months. Specifically, the time between session 1 and session 2 was of mean = 2.75 (1.31) months; mean = 4.54 (1.64) months between sessions 2 and 3; and mean = 3.91 (1.36) months between sessions 3 and 4. The longest interval was between sessions 2 and 3 because of the summer holidays.

#### Covariates

Socio-demographic characteristics including child date of birth, gender, maternal education level (primary or less, secondary and university) and home addresses were obtained from a questionnaire completed by parents during 2012. Children's age for each session was calculated based on birth date and session date. A neighborhood socioeconomic status vulnerability index (based on level of education, unemployment, and occupation at the census tract (Sunyer et al., 2015) was calculated at the home address.

Teachers completed an ADHD symptoms questionnaire based on the diagnostic criteria for ADHD as described in the Diagnostic and Statistical Manual of Mental Disorders, fourth Edition (ADHD-DSM-IV; American Psychiatric Association, 2000). ADHD-DSM-IV consists of a list of 18 symptoms categorized under two separate symptom groups (inattention and hyperactivity/impulsivity) with nine symptoms each. Each ADHD symptom is rated on a 4-point scale (0 never or rarely, 1 sometimes, 2 often, 3 very often). We recoded options 0 and 1 as 0 (symptom absent), and options 2 and 3 as 1 (symptom present) (Gomez, 2007). We used a categorical variable of ADHD clinical criteria with four categories, according to the presence of 6 or more symptoms of each subtype: no ADHD; ADHD-inattentive; ADHD-hyperactive/impulsive; and ADHD-combined. The teacher ratings of ADHD symptoms used in this study are not to be confounded with a clinical diagnosis of ADHD by a medical doctor.

### Statistical analysis

Due to the hierarchical structure of the data (children embedded within schools and repeated measures collected on a child over time), we used multilevel mixed-effects linear regression models for each outcome to study the developmental trajectories across sessions. We included random intercepts by school and individual and random slopes by individual for the linear and quadratic effects of age (included to capture the nonlinearity in the growth trajectories of attention, if any). The equation of the model was the following,

$$\begin{array}{l} {\text{Y}}_{\text{sit}}=\left(\beta_{0}+{\text{u}}_{0\text{s}}+{\text{s}}_{0\text{i}\left(\text{s}\right)}\right)+{\left(\beta_{1}+{\text{s}}_{1\text{i}\left(\text{s}\right)}\right)}^{*}{\text{age}}_{\text{sit}}\\ +{\left(\beta_{2}+s_{2\text{i}\left(\text{s}\right)}\right)}^{*}{\text{age}}_{\text{sit}}^{2}+\varepsilon_{\text{sit},} \end{array}$$

Where Y_sit_ is the ANT outcome for individual *i* within school *s* at session *t, t* = {1,2,3,4}, u_*s*_ are random effects at school level, s_i(s)_ are random effects associated with the individual *i* within school s, and ε_sit_ are the residuals.

First, random effects associated with age were tested using likelihood-ratio tests. Afterwards, we included the interaction between age and gender, and stratified models were presented if the growth pattern differed according to gender. Then, we tested interactions between age and teacher-rated ADHD symptoms, and the models were stratified when the interactions were statistically significant. Fixed effects were tested using Wald tests. To visualize the shape of the growth function, we plotted the average predicted curve and 95% confidence band.

Statistical significance was set at *p* < 0.05. Statistical analyses were done using R (3.0.2; R Foundation for Statistical Computing) and Stata 12.1 (Stata Corporation, College Station, Texas).

## Results

Children were on average 8.6 years old at baseline and 49.6% were girls. Maternal education level was high (58.9% of mothers had a university degree). According to the questionnaires rated by the teachers 10.5% of children presented ADHD symptoms, being the inattentive subtype the most prevalent across age groups (Table 2).

**Table 2:** **Demographic and clinical characteristics of the sample by grade (*n* = 2835)**.

	**2nd grade (*n* = 1064)**	**3rd grade (*n* = 1015)**	**4th grade (*n* = 756)**
Age at session 1 (mean, SD)	7.7 (0.33)	8.7 (0.37)	9.7 (0.36)
Gender (% girls)	49.0	48.9	51.5
Home socioeconomic vulnerability index (mean, SD)	0.45 (0.20)	0.45 (0.21)	0.45 (0.21)
**Maternal education (%)**			
Primary or less	15.2	11.0	11.8
Secondary	24.7	28.0	34.0
University	60.1	61.0	54.2
**ADHD subtypes -DSM-IV, teachers form^a^ (%)**			
No-ADHD	90.0	90.6	88.0
ADHD-inattentive	6.13	6.18	6.41
ADHD-hyperactive/impulsive	1.1	1.6	2.5
ADHD-combined	2.7	1.6	3.1

*ADHD, Attention Deficit Hyperactivity Disorder*.

a *ADHD Criteria of Diagnostic and Statistical Manual of Mental Disorders, fourth edition. Teachers form*.

In the multilevel mixed-effects linear regression models we found improvements related to age for all the outcomes, except orienting. Gender interacted with age in executive attention (*p* for interaction < 0.001) and HRT (*p* for interaction < 0.001). Teacher-rated ADHD symptoms interacted with age in executive attention (*p* for interaction = 0.027), HRT (*p* for interaction = 0.014) and variability (*p* for interaction = 0.009) (Table 3).

**Table 3:** **Age-associated changes (coefficient, 95% CI)^†^ in the ANT outcomes during the 1-year follow-up**.

**Outcome**	**Age**	***p***	**Age^2^^¶^**	***p***
**ALERTING**				
All	4.65 (−1.26, 10.57)	0.123	−1.44 (−2.84, −0.04)	0.043
**ORIENTING**				
All	−1.47 (−2.96, 0.02)	0.053	–	
**EXECUTIVE ATTENTION**				
Boys	−17.30 (−23.96, −10.64)	<0.001	2.19 (0.61, 3.78)	0.007
Girls	−4.19 (−5.88, −2.50)	<0.001	–	
No ADHD	−6.02 (−7.28, −4.77)	<0.001	–	
ADHD-inattentive	−10.69 (−16.69, −4.68)	<0.001	–	
ADHD-hyperactive-impulsive	−3.89 (−13.44, 5.64)	0.423	–	
ADHD-combined	−16.55 (−29.20, −3.91)	0.010	–	
**HRT**				
Boys	−154.37 (−168.26, −140.48)	<0.001	15.62 (12.36, 18.88)	<0.001
Girls	−185.56 (−200.70, −170.43)	<0.001	19.37 (15.89, 22.84)	<0.001
No ADHD	−171.15 (−182.01, −160.29)	<0.001	17.59 (15.06, 20.12)	<0.001
ADHD-inattentive	−197.90 (−240.19, −155.61)	<0.001	22.16 (12.52, 31.80)	<0.001
ADHD-hyperactive-impulsive	−58.24 (−80.30, −36.18)	<0.001	–	
ADHD-combined	−79.96 (−104.23, −55.68)	<0.001	–	
**HRT-SE**				
All	−62.26 (−69.44, −55.09)	<0.001	7.50 (5.82, 9.18)	<0.001
**VARIABILITY**				
No ADHD	−2.93 (−3.86, −2.01)	<0.001	–	
ADHD-inattentive	1.24 (−2.12, 4.60)	0.469	–	
ADHD-hyperactive-impulsive	−8.83 (−15.65, −2.01)	0.011	–	
ADHD-combined	−6.88 (−13.28, −0.49)	0.035	–	

*CI, Confidence Interval, RT, Reaction Time, SE, Standard Error, ADHD, Attention Deficit and Hyperactivity Disorder*.

† *Coefficients obtained from multilevel mixed-effects linear regression models including school, individual and age as nested random effects. Stratified results by gender and ADHD symptoms are provided when p-value for interaction ≤0.05*.

¶ *When the association with age was not linear, a quadratic function was fitted*.

*–No effect*.

*“All” refers to all children*.

**Figure 1:**
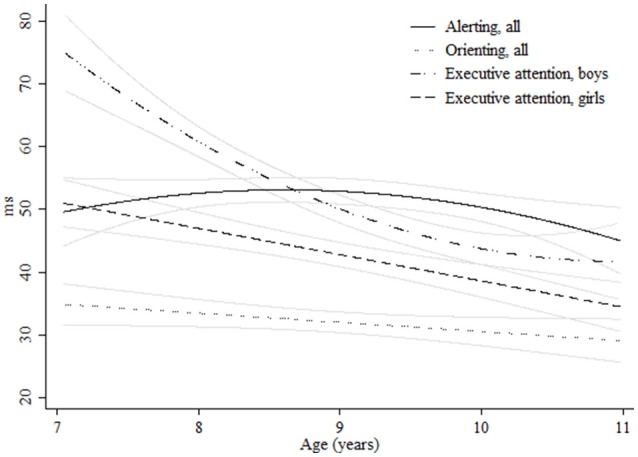
**Age-associated predicted curves for the network scores**. Average predicted curves and 95% confidence bands. Stratified results by gender are provided for executive attention.

**Figure 2:**
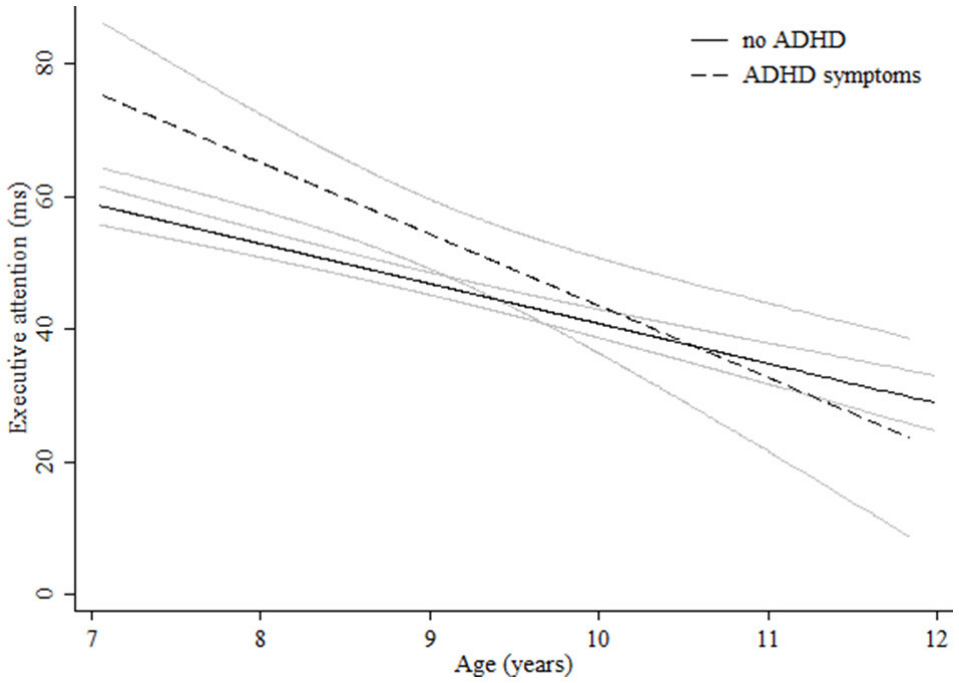
**Age-associated predicted curves for executive attention**. Average predicted curves and 95% confidence bands. Stratified results by ADHD symptoms are provided.

**Figure 3:**
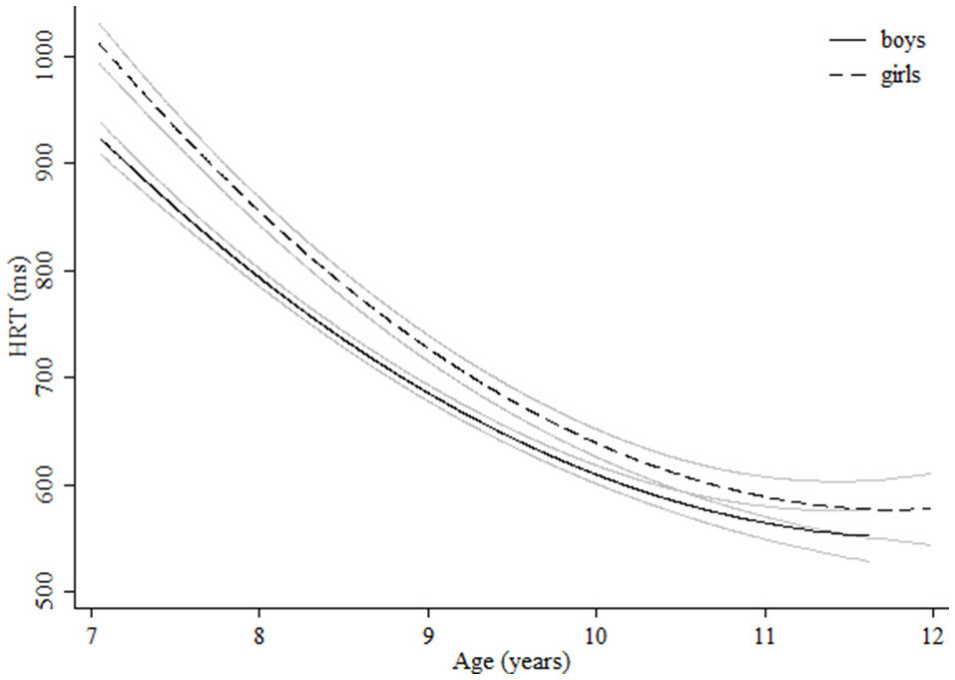
**Age-associated predicted curves for HRT**. Average predicted curves and 95% confidence bands. Stratified results by gender are provided.

**Figure 4:**
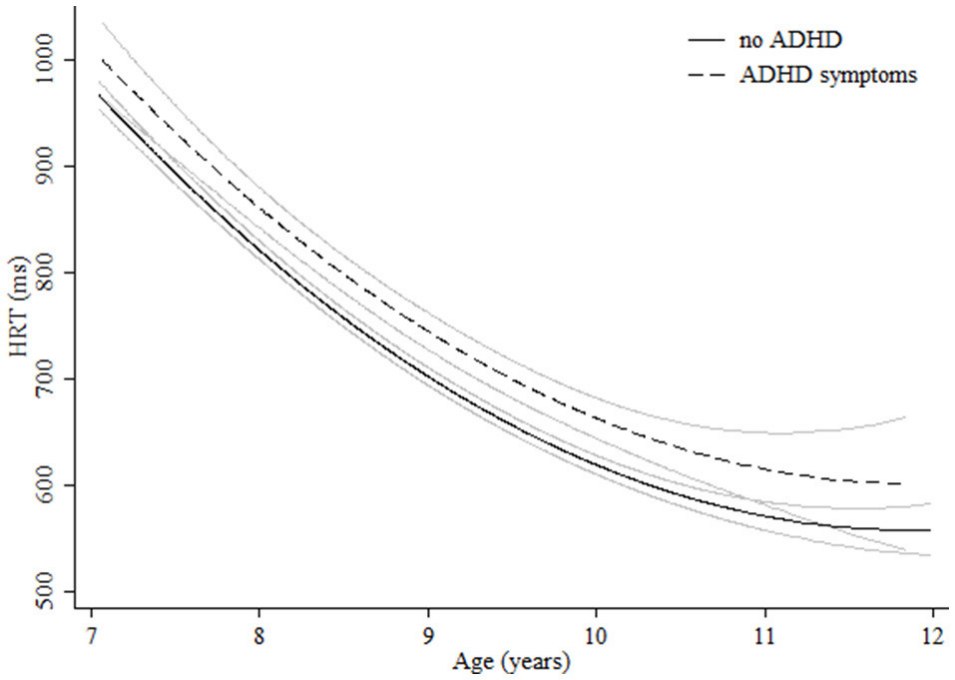
**Age-associated predicted curves for HRT**. Average predicted curves and 95% confidence bands. Stratified results by ADHD symptoms are provided.

**Figure 5:**
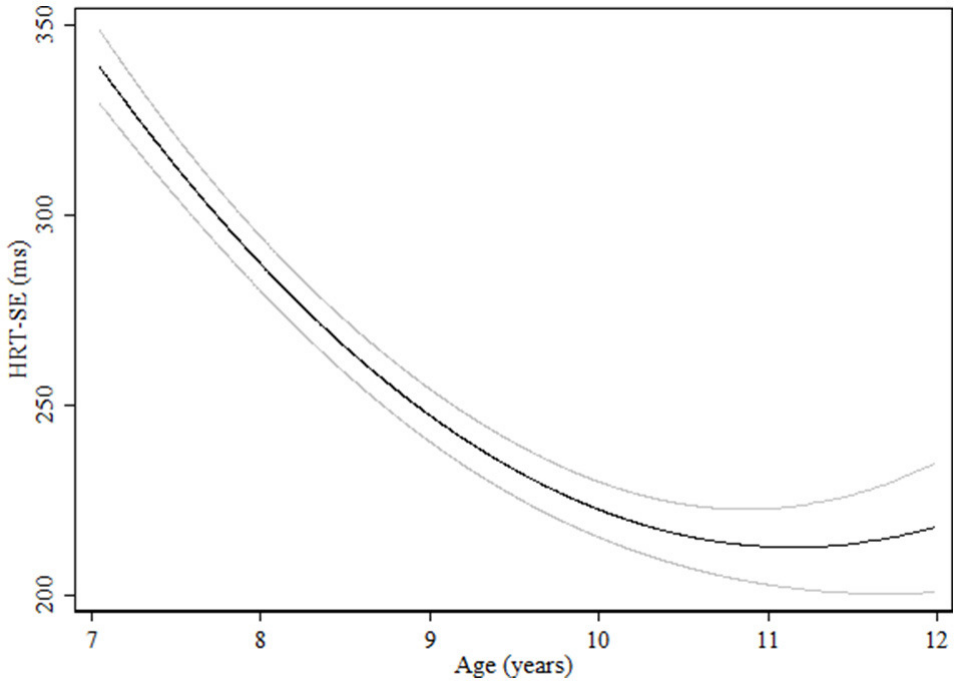
**Age-associated predicted curve for HRT-SE**. Average predicted curve and 95% confidence band.

**Figure 6:**
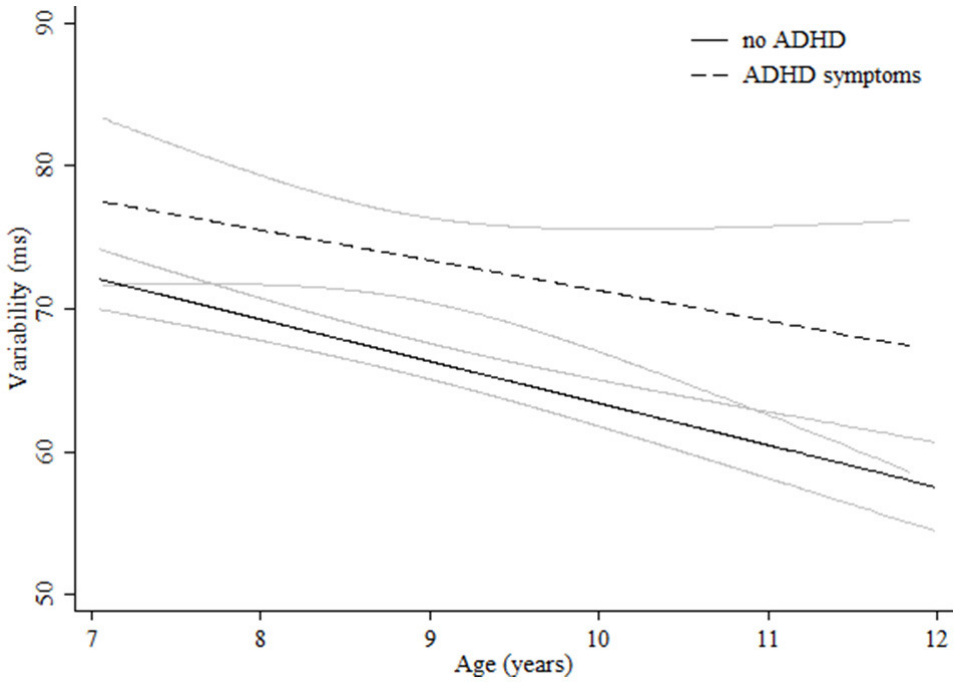
**Age-associated predicted curves for Variability**. Average predicted curves and 95% confidence bands. Stratified results by ADHD symptoms are provided.

The inclusion of maternal education and socioeconomic status in the models did not change the results (Table S2). Furthermore, the interactions between age and maternal education (*p* for interaction = 0.263) and socioeconomic status (*p* for interaction = 0.093) were unrelated to executive attention development (Table S3). Figures 1–**6** represent age-associated changes in the ANT networks scores and measures related to attentiveness.

Regarding the network scores, for alerting we detected a quadratic curve indicating a further growth for the oldest ages (Figure 1, Table 3). Different age-related patterns by gender or ADHD symptoms were not found. We obtained no significant age effect for orienting (Figure 1, Table 3). For executive attention, girls performed better at young ages. However, males showed a faster cognitive growth and reached girls performance at around 10 years of age (Figure 1). Children with teacher-rated ADHD symptoms, specifically the inattentive and combined subtypes, performed worse than children with no ADHD symptoms until age 9 in executive attention. No age-related changes were encountered in children with ADHD hyperactive-impulsive symptoms (Figure 2, Table 3).

Regarding measures related to attentiveness, for HRT quadratic curves were found indicating more pronounced increases in performance in the younger groups (Table 3). Although boys were significantly quicker, girls showed a more pronounced improvement during the age range studied and reached the levels of boys at age 11 (Figure 3). Children with ADHD symptoms showed quicker HRT across the entire age range but without reaching the levels of children with no ADHD symptoms (Figure 4, Table 3). For HRT-SE, we found a quadratic curve, indicating a greater improvement in the younger groups that stabilized at around 10 years of age (Figure 5). Different age-related patterns by gender or ADHD symptoms were not detected. Finally, for variability different age-related patterns by gender were not detected. Children with ADHD hyperactive-impulsive and combined symptoms showed higher variability than those without ADHD symptoms across all age groups (Table 3, Figure 6). No age-related changes were encountered in children with ADHD inattention symptoms (Table 3).

## Discussion

The present study explored, for the first time, the attention development using the ANT in a large sample (*n* = 2,835) of primary-school children in a prospective longitudinal study using repeated measures. The pathways of the three attention networks and measures related to attentiveness showed different developmental trajectories. Specifically, the attention development in the age range studied was more pronounced in executive attention, HRT, HRT-SE, and variability, particularly from 7 to 9 years of age. We detected gender differences in executive attention and HRT up to age 10. Children with teacher-rated ADHD symptoms showed a developmental delay in executive attention, HRT and variability.

The trajectories of attention growth observed confirm previous cross-sectional studies that used the ANT, which described an ongoing development of some aspects of attention during early and late childhood (i.e., ages 5–12) (Mezzacappa, 2004; Rueda et al., 2004; Mullane et al., 2016; Federico et al., 2017). This is consistent with neuroimaging studies reporting continued myelinization of the neural circuitry involved in attention processes until adolescence (Hudspeth and Pribram, 1992; Konrad et al., 2005). Furthermore, larger improvements in the younger groups correlate with the fourth rapid brain growth stage (i.e., ages between 6 and 8 years; Epstein, 1986).

Regarding the developmental trajectories of the attention networks, for alerting we found a slow progression toward the end of the investigated age range (Ridderinkhof et al., 1997; Gupta and Kar, 2009; Mullane et al., 2016; Federico et al., 2017). Alertness starts to develop in the first few postnatal weeks and by the third month babies are able to maintain an alert state in relation to the external sensory stimulation (Rueda et al., 2015). Despite the presence in infancy, the alerting network undergoes a significant improvement during late childhood in relation to continued development of frontal regions during this period (Rueda et al., 2004). The orienting network starts developing very early in infancy; newborns show head an eye movements toward a peripheral cue and from age 4 months there is great stability (Clohessy et al., 2001; Rueda et al., 2004; Gupta and Kar, 2009). For orienting we obtained no significant age changes in children from 7 to 11 years old, consistent with the formerly described early maturation of this network. The executive attention network follows a more prolonged development during childhood and into early adolescence (Ridderinkhof et al., 1997; Rueda et al., 2004; van Meel et al., 2012; Loher and Roebers, 2013; Mullane et al., 2016). Executive attention involves detecting and resolving conflict among responses, error detection and response inhibition. Its development is related to the maturation of the anterior cingulate cortex and lateral prefrontal cortex (Bush et al., 2000) which are not fully matured until adolescence (Romine and Reynolds, 2005). Furthermore, we encountered that the developmental course of the executive attention network differed by gender. Girls were superior compared to boys in the younger groups, probably reflecting variations in the maturation rate between males and females up to ages of 9 and 11 years (Pascualvaca et al., 1997). A more rapid biological, cognitive, and social-emotional development of girls is in fact well-known (Keenan and Shaw, 1997; Cahill, 2006). Finally, in our study the executive attention trajectory was unrelated to maternal education or socioeconomic status, in contrast to previous literature documenting an association between different indicators of socioeconomic status and executive function development (Hackman and Farah, 2009).

Regarding the measures related to attentiveness, significant improvements in reaction time (HRT), response speed consistency (HRT-SE) and intra-individual variability were found over the age range studied, and particularly between ages 7 and 9 years (Levy, 1980; Rebok et al., 1997). Boys had faster reaction times compared to girls (Pascualvaca et al., 1997; Klenberg et al., 2001), although the progression was further for females. The lack of significant differences in the trajectories of response speed consistency and intra-individual variability by gender, suggests similar age trends for males and females for these aspects of attention (Pascualvaca et al., 1997).

The developmental trajectories for executive attention, response speed and intra-individual variability were significantly different in children with teacher-rated ADHD symptoms compared to typically developing children. Neuroimaging studies have reported a delay of 2–3 years in brain maturation in ADHD, and not a complete deviation from typical development (Castellanos et al., 2002; Shaw et al., 2007). Difficulties in executive attention and alerting networks have been described as deficits that underlie ADHD (Berger and Posner, 2000). As expected, typically developing children outperformed children with ADHD symptoms in the executive attention network which involves response inhibition, indeed the essential impairment in this disorder (Barkley, 1997). However, we encountered no differences in the developmental trajectory of alerting in the age range studied between the two groups. The late further development of the alerting network (e.g., from 10 years of age) (Ridderinkhof et al., 1997; Rueda et al., 2004) may partly explain these results. Furthermore, in the published literature there are also discrepancies related to a weaker alertness in ADHD (Booth et al., 2007; Adólfsdóttir et al., 2008; Johnson et al., 2008; Gupta and Kar, 2009; Mullane et al., 2011; Casagrande et al., 2012). Regarding reaction time in responding to a target, slower response speed has been reported previously for children with ADHD (Gupta and Kar, 2009; Mullane et al., 2011), and that is in fact what we observed in our study. Children with ADHD symptoms had a developmental trajectory for response speed that paralleled the growth curve for typically developing children but on a lower track. Finally, in relation to intra-individual variability, we encountered more variability in response speed consistency in children with ADHD symptoms compared to children without ADHD symptoms. The moment-to-moment fluctuations in attention is in fact the most remarkable symptom of ADHD (Castellanos and Tannock, 2002). Indeed, intra-individual response variability measures, defined as short-term fluctuations in performance of an individual over a time-scales of seconds, have been described as the best to discriminate between ADHD and control groups (Russell et al., 2006). Finally, in relation to ADHD subtypes, no clear differential patterns of attention trajectories were observed despite the well-described clinical differences between them (Cantwell and Baker, 1992).

This study has some strengths and limitations that need to be considered. The present findings are dependent on the version of the ANT used, the age range of the participants and the teacher-rated ADHD symptoms—not a medical diagnosis of ADHD. However, we found that the child ANT (Rueda et al., 2004) was able to detect attention trajectories in childhood, including gender differences and ADHD symptoms. In relation to the age effects, the late development of the alerting network or the earlier executive attention development in females, for instance, still needs further investigation. Furthermore, the normalization of the performance in executive attention at around 10 years of age in children with teacher-rated ADHD symptoms needs future confirmation. Further studies using the original ANT (Fan et al., 2002) are warranted in order to discard any ceiling effects of the child ANT in late childhood. Finally, the cognitive change observed with repeated test application over a 1-year period may still include some practice effects. However, in previous studies, despite short test-retest intervals (i.e., hours or days) in attentional function in children or in adults the magnitude of the practice effects was moderate to small and the ANT networks showed robustness against practice (Mollica et al., 2005; Ishigami and Klein, 2011). Furthermore, the exact age in each assessment and the time intervals between the neuropsychological testing varied among the children and that contributed to minimizing practice effects. Strengths of this population-based study include the large sample size and the longitudinal design for the detection of cognitive change. Repeated measurements within-participants provided the prospective data required to define developmental trajectories. In addition to the attention networks of the ANT, three measures related to attentiveness were also calculated and allowed us to explore the growth patterns of more aspects of attention. Furthermore, we analyzed the role of gender and ADHD symptoms in attention development. Finally, the study of the attention development in grade-schoolers can be used to identify students who deviate from normality and the identification of possible environmental or social risk factors. This would contribute to the implementation of school accommodations or other interventions needed for these children. The implications of attentional deficits in the school setting are non-negligible since they are associated with behavioral difficulties and poor academic achievement (e.g., lack of self-control, failure to complete tasks, and commission of procedural mistakes) (Pascualvaca et al., 1997; Anderson, 2002).

In summary, we observed an ongoing development of some aspects of attention in primary school aged children. Nevertheless, the developmental changes were more evident in executive attention and measures related to attentiveness, and in the younger groups. Furthermore, girls were more advantaged at younger ages and children with teacher-rated ADHD symptoms showed a delayed development in some attention processes. Our findings support the ANT for assessing attention in children in large epidemiological studies.

## Author contributions

ES, JF, and JS conceptualized and designed the study and drafted the initial manuscript. RG, ME, and XB supported and supervised the statistical analyses and revised the manuscript. MA coordinated and supervised data collections and critically reviewed the manuscript. ML, AL, JJ, and AC supervised the interpretation of the results and critically reviewed the manuscript.

## Funding

The research leading to these results received funding from the European Research Council under the ERC Grant Agreement number 268479 – the BREATHE project. JJ holds a Miguel Servet contract (MS14/00108) awarded by the Spanish Institute of Health Carlos III (Ministry of Economy and Competitiveness).

### Conflict of interest statement

The authors declare that the research was conducted in the absence of any commercial or financial relationships that could be construed as a potential conflict of interest.
