# Supplementary material for: A Longitudinal Study on Attention Development in Primary School Children with and without Teacher-Reported Symptoms of ADHD
Source: Front Psychol. 2017 May 16;8:655. doi: 10.3389/fpsyg.2017.00655 (PMC5432613; doi:10.3389/fpsyg.2017.00655)
Supplement: Supplementary file 1 [file Table1.DOCX]

| \| \| **TABLE S1. Age-associated changes (coefficient, 95% CI)^†^ in the ANT outcomes during the 1-year follow-up.** \| \| \| \| \| \| --- \| --- \| --- \| --- \| --- \| \| **Outcome** \| **Age** \| ***p*** \| **Age^2 ¶^** \| ***p*** \| \| **Alerting** \|  \|  \|  \|  \| \| All \| 2.03 (-4.15, 8.22) \| 0.519 \| -0.89 (-2.36, 0.57) \| 0.231 \| \| **Orienting** \|  \|  \|  \|  \| \| All \| -1.34 (-2.98, 0.29) \| 0.109 \| – \|  \| \| **Executive attention** \|  \|  \|  \|  \| \| Boys \| -15.14 (-22.05, -8.23) \| <0.001 \| 1.60 (-0.03, 3.24) \| 0.055 \| \| Girls \| -4.52 (-6.35, -2.68) \| <0.001 \| – \|  \| \| No ADHD \| -5.99 (-7.37, -4.62) \| <0.001 \| – \|  \| \| ADHD-inattentive \| -12.16 (-19.26, -5.06) \| 0.001 \| – \|  \| \| ADHD-hyperactive-impulsive \| -10.37 (-21.54, 0.81) \| 0.069 \| – \|  \| \| ADHD-combined \| -17.76 (-27.91, -7.61) \| 0.001 \| – \|  \| \| **HRT** \|  \|  \|  \|  \| \| Boys \| -145.20 (-160.06, -130.34) \| <0.001 \| 13.14 (9.70, 16.59) \| <0.001 \| \| Girls \| -179.73 (-195.85, -163.61) \| <0.001 \| 17.52 (13.82, 21.21) \| <0.001 \| \| No ADHD \| -161.22 (-172.84, -149.59) \| <0.001 \| 14.91 (12.23, 17.59) \| <0.001 \| \| ADHD-inattentive \| -194.42 (-240.75, -148.09) \| <0.001 \| 21.08 (10.51, 31.65) \| <0.001 \| \| ADHD-hyperactive-impulsive \| -69.28 (-96.15, -42.40) \| <0.001 \| – \|  \| \| ADHD-combined \| -81.95 (-106.49, -57.42) \| <0.001 \| – \|  \| \| **HRT-SE** \|  \|  \|  \|  \| \| All \| -67.38 (-75.14, -59.62) \| <0.001 \| 8.18 (6.37, 9.99) \| <0.001 \| \| **Variability** \|  \|  \|  \|  \| \| No ADHD \| -3.46 (-4.47, -2.46) \| <0.001 \| – \|  \| \| ADHD-inattentive \| 0.88 (-2.95, 4.72) \| 0.653 \| – \|  \| \| ADHD-hyperactive-impulsive \| -9.05 (-16.52, -1.55) \| 0.018 \| – \|  \| \| ADHD-combined \| -10.19 (-17.62, -2.76) \| 0.007 \| – \|  \| \| *CI, Confidence Interval, RT, Reaction Time, SE, Standard Error, ADHD, Attention Deficit and Hyperactivity Disorder.* \| \| \| \| \| \| \| *† Coefficients obtained from multilevel mixed-effects linear regression models adjusted for environmental factors (day of the week, season, noise, weather, time of the day, and quality of the session); school, individual and age as nested random effects. Stratified results by gender and ADHD symptoms are provided when p-value for interaction ≤0.05.* \| \| \| \| \| \| *^¶^ When the association with age was not linear, a quadratic function was fitted.* \| \| \| \| \| *– No effect.* \|  \|  \|  \|   *“All” refers to all children.*  **TABLE S2. Age-associated changes (coefficient, 95% CI)^†^ in the ANT outcomes during the 1-year follow-up.** \| \| \| \| \| \| --- \| --- \| --- \| --- \| --- \| --- \| --- \| --- \| --- \| --- \| --- \| --- \| --- \| --- \| --- \| --- \| --- \| --- \| --- \| --- \| --- \| --- \| --- \| --- \| --- \| --- \| --- \| --- \| --- \| --- \| --- \| --- \| --- \| --- \| --- \| --- \| --- \| --- \| --- \| --- \| --- \| --- \| --- \| --- \| --- \| --- \| --- \| --- \| --- \| --- \| --- \| --- \| --- \| --- \| --- \| --- \| --- \| --- \| --- \| --- \| --- \| --- \| --- \| --- \| --- \| --- \| --- \| --- \| --- \| --- \| --- \| --- \| --- \| --- \| --- \| --- \| --- \| --- \| --- \| --- \| --- \| --- \| --- \| --- \| --- \| --- \| --- \| --- \| --- \| --- \| --- \| --- \| --- \| --- \| --- \| --- \| --- \| --- \| --- \| --- \| --- \| --- \| --- \| --- \| --- \| --- \| --- \| --- \| --- \| --- \| --- \| --- \| --- \| --- \| --- \| --- \| --- \| --- \| --- \| --- \| --- \| --- \| --- \| --- \| --- \| --- \| --- \| --- \| --- \| --- \| --- \| --- \| --- \| --- \| --- \| --- \| --- \| --- \| --- \| --- \| --- \| --- \| --- \| --- \| --- \| --- \| --- \| --- \| --- \| --- \| --- \| --- \| --- \| --- \| --- \| --- \| --- \| --- \| --- \| \| **Outcome** \| **Age** \| ***p*** \| **Age^2 ¶^** \| ***p*** \| \| **Alerting** \|  \|  \|  \|  \| \| All \| 4.82 (-1.21, 10.86) \| 0.117 \| -1.51 (-2.94, -0.08) \| 0.039 \| \| **Orienting** \|  \|  \|  \|  \| \| All \| -1.39 (-2.92, 0.14) \| 0.075 \| – \|  \| \| **Executive attention** \|  \|  \|  \|  \| \| Boys \| -16.79 (-23.60, -9.98) \| <0.001 \| 2.14 (0.51, 3.77) \| 0.010 \| \| Girls \| -3.92 (-5.64, -2.21) \| <0.001 \| – \|  \| \| No ADHD \| -5.75 (-7.02, -4.47) \| <0.001 \| – \|  \| \| ADHD-inattentive \| -9.12 (-15.02, -3.22) \| 0.002 \| – \|  \| \| ADHD-hyperactive-impulsive \| -5.84 (-15.89, 4.20) \| 0.254 \| – \|  \| \| ADHD-combined \| -17.73 (-30.45, -5.02) \| 0.006 \| – \|  \| \| **HRT** \|  \|  \|  \|  \| \| Boys \| -154.54 (-168.74, -140.34) \| <0.001 \| 15.86 (12.50, 19.21) \| <0.001 \| \| Girls \| -183.12 (-198.43, -167.82) \| <0.001 \| 18.88 (15.36, 22.40) \| <0.001 \| \| No ADHD \| -169.10 (-180.12, -158.07) \| <0.001 \| 17.23 (14.66, 19.80) \| <0.001 \| \| ADHD-inattentive \| -199.17 (-242.79, -155.55) \| <0.001 \| 22.57 (12.53, 32.61) \| <0.001 \| \| ADHD-hyperactive-impulsive \| -59.90 (-82.81, -36.98) \| <0.001 \| – \|  \| \| ADHD-combined \| -78.15 (-102.41, -53.89) \| <0.001 \| – \|  \| \| **HRT-SE** \|  \|  \|  \|  \| \| All \| -61.62 (-68.91, -54.34) \| <0.001 \| 7.41 (5.70, 9.12) \| <0.001 \| \| **Variability** \|  \|  \|  \|  \| \| No ADHD \| -3.14 (-4.07, -2.20) \| <0.001 \| – \|  \| \| ADHD-inattentive \| 1.38 (-2.09, 4.84) \| 0.436 \| – \|  \| \| ADHD-hyperactive-impulsive \| -7.96 (-14.87, -1.04) \| 0.024 \| – \|  \| \| ADHD-combined \| -7.61 (-14.10, -1.11) \| 0.022 \| – \|  \| \| *CI, Confidence Interval, RT, Reaction Time, SE, Standard Error, ADHD, Attention Deficit and Hyperactivity Disorder.* \| \| \| \| \| \| *† Coefficients obtained from multilevel mixed-effects linear regression models adjusted for maternal education and socioeconomic status; school, individual and age as nested random effects. Stratified results by gender and ADHD symptoms are provided when p-value for interaction ≤0.05.* \| \| \| \| \| \| *^¶^ When the association with age was not linear, a quadratic function was fitted.* \| \| \| \|  \| \| *– No effect.* \|  \|  \|  \|  \|   *“All” refers to all children.*  **Table S3. Age-associated changes (coefficient, 95% CI) in Executive attention during the 1-year follow-up by maternal education level and socioeconomic status.**   \|  \| **Age** \| **Age^2 ¶^** \| ***p-value***  **interaction** \| \| --- \| --- \| --- \| --- \| \| **Maternal education level** \|  \|  \| 0.263 \| \| Primary or less (n = 344) \| -6.09 (-10.03, -2.15) \| – \|  \| \| Secondary (n = 764) \| -6.93 (-9.37, -4.48) \| – \|  \| \| University (n = 1589) \| -6.01 (-7.56, -4.46) \| – \|  \| \|  \|  \|  \|  \| \| **Socioeconomic status** (tertiles) \|  \|  \| 0.093 \| \| Low (n = 1059) \| -7.76 (-9.65, -5.88) \| – \|  \| \| Medium (n = 883) \| -14.64 (-22.92, -6.36) \| 2.03 (0.06, 3.99) \|  \| \| High (n = 885) \| -4.70 (-7.09, -2.31) \| – \|  \|   *CI, Confidence Interval.*  *^¶^ When the association with age was not linear, a quadratic function was fitted.*  *– No effect.* |
| --- | --- | --- | --- | --- | --- | --- | --- | --- | --- | --- | --- | --- | --- | --- | --- | --- | --- | --- | --- | --- | --- | --- | --- | --- | --- | --- | --- | --- | --- | --- | --- | --- | --- | --- | --- | --- | --- | --- | --- | --- | --- | --- | --- | --- | --- | --- | --- | --- | --- | --- | --- | --- | --- | --- | --- | --- | --- | --- | --- | --- | --- | --- | --- | --- | --- | --- | --- | --- | --- | --- | --- | --- | --- | --- | --- | --- | --- | --- | --- | --- | --- | --- | --- | --- | --- | --- | --- | --- | --- | --- | --- | --- | --- | --- | --- | --- | --- | --- | --- | --- | --- | --- | --- | --- | --- | --- | --- | --- | --- | --- | --- | --- | --- | --- | --- | --- | --- | --- | --- | --- | --- | --- | --- | --- | --- | --- | --- | --- | --- | --- | --- | --- | --- | --- | --- | --- | --- | --- | --- | --- | --- | --- | --- | --- | --- | --- | --- | --- | --- | --- | --- | --- | --- | --- | --- | --- | --- | --- | --- | --- | --- | --- | --- | --- | --- | --- | --- | --- | --- | --- | --- | --- | --- | --- | --- | --- | --- | --- | --- | --- | --- | --- | --- | --- | --- | --- | --- | --- | --- | --- | --- | --- | --- | --- | --- | --- | --- | --- | --- | --- | --- | --- | --- | --- | --- | --- | --- | --- | --- | --- | --- | --- | --- | --- | --- | --- | --- | --- | --- | --- | --- | --- | --- | --- | --- | --- | --- | --- | --- | --- | --- | --- | --- | --- | --- | --- | --- | --- | --- | --- | --- | --- | --- | --- | --- | --- | --- | --- | --- | --- | --- | --- | --- | --- | --- | --- | --- | --- | --- | --- | --- | --- | --- | --- | --- | --- | --- | --- | --- | --- | --- | --- | --- | --- | --- | --- | --- | --- | --- | --- | --- | --- | --- | --- | --- | --- | --- | --- | --- | --- | --- | --- | --- | --- | --- | --- | --- | --- | --- | --- | --- | --- | --- | --- | --- | --- | --- | --- | --- | --- | --- | --- | --- | --- | --- | --- | --- | --- | --- | --- | --- | --- | --- | --- | --- | --- | --- | --- | --- | --- | --- | --- | --- | --- | --- | --- | --- | --- | --- | --- | --- | --- | --- | --- | --- | --- | --- | --- | --- |
